# Cooking Effect on the Bioactive Compounds, Texture, and Color Properties of Cold-Extruded Rice/Bean-Based Pasta Supplemented with Whole Carob Fruit

**DOI:** 10.3390/foods9040415

**Published:** 2020-04-02

**Authors:** Claudia Arribas, Blanca Cabellos, Carmen Cuadrado, Eva Guillamón, Mercedes M. Pedrosa

**Affiliations:** 1 Food Technology Department, SGIT-INIA, Ctra de La Coruña, Km 7.5, 28040 Madrid, Spain; correoinia@gmail.com (C.A.); blancacc@inia.es (B.C.); cuadrado@inia.es (C.C.); 2 Centre for the Food Quality, SGIT-INIA, C/Universidad s/n, 42004 Soria, Spain; guillamon.eva@inia.es

**Keywords:** prebiotics, trypsin inhibitors, inositol phosphates, phenols, legumes, functional foods, gluten-free

## Abstract

Pasta is considered as the ideal vehicle for fortification; thus, different formulations of gluten-free pasta have been developed (rice 0–100%, bean 0–100%, and carob fruit 0% or 10%). In this article, the content of individual inositol phosphates, soluble sugars and α-galactosides, protease inhibitors, lectin, phenolic composition, color, and texture were determined in uncooked and cooked pasta. The highest total inositol phosphates and protease inhibitors contents were found in the samples with a higher bean percentage. After cooking, the content of total inositol phosphates ranged from 2.12 to 7.97 mg/g (phytic acid or inositol hexaphosphate (IP6) was the major isoform found); the protease inhibitor activities showed values up to 12.12 trypsin inhibitor (TIU)/mg and 16.62 chymotrypsin inhibitor (CIU)/mg, whereas the competitive enzyme-linked immunosorbent assay (ELISA) showed the elimination of lectins. Considering the different α-galactosides analyzed, their content was reduced up to 70% (*p* < 0.05) by the cooking process. The total phenols content was reduced around 17–48% after cooking. The cooked samples fortified with 10% carob fruit resulted in darker fettuccine with good firmness and hardness and higher antioxidant activity, sucrose, and total phenols content than the corresponding counterparts without this flour. All of the experimental fettuccine can be considered as functional and healthy pasta mainly due to their bioactive compound content, compared to the commercial rice pasta.

## 1. Introduction

Pasta is largely consumed all over the world and, moreover, these kinds of products are considered by the World Health Organization (WHO) as an ideal vehicle for fortification [1]. Pasta is a cereal-based product characterized by good organoleptic and nutritional properties and long shelf life. Pasta, such as spaghetti and macaroni, are manufactured traditionally by mixing durum wheat semolina and water (and eggs in some types of products). During the wheat pasta-making process, gluten is the principal compound responsible for the formation of the pasta structure, and it provides better quality parameters (low loss of solids in the cooking process, firm structure, attractive color, etc.) than those of gluten-free (GF) pasta mainly elaborated with corn, sorghum, rice, and/or legumes [2]. Nowadays, the population with celiac disease, as well as those who exclude the gluten-containing traditional products from their diet for other health reasons, is growing; therefore, there is a strong need for the development of novel nutritious and healthy products. 

In order to elaborate GF pasta, the gluten matrix of the traditional pasta products can be substituted by other starchy raw materials and/or new formulations, alternative pasta-making processes, and/or the addition of additives; the raw materials and the pasta-making process have a great effect on the quality of the pasta [3]. The use of starchy cereals, such as rice, combined with pulses can provide nutritive and balanced pasta products, with a good content and profile of proteins, dietary fiber, complex carbohydrates, minerals, and vitamins [4,5]. As reported by Elliott et al. [6], GF products are not nutritionally finer than gluten-containing products. Thus, these new pasta products based on cereal and pulse mixtures would be of great interest to celiac patients, as well as to those people that decide to eliminate gluten from their diets. White rice is the main ingredient used by the food industry to manufacture GF products, even though it is a material with a poor bioactive compound content. On the contrary, pulses are rich in bioactive compounds (e.g., phenolic compounds, phytates, saponins, α-galactosides, or trypsin inhibitors). Traditionally, some of these phytochemicals such as α-galactosides, trypsin inhibitors, or phytates have been considered as anti-nutritive compounds that can produce flatulence, reduce protein digestion, or impair the absorption of other compounds, respectively. However, at present, it is well known that these same compounds are related to a reduction in the risk of cardiovascular disease, type 2 diabetes, digestive tract diseases, becoming overweight, and obesity [5]. Therefore, the fortification of rice-based pasta with legumes would increase the amount of bioactive compounds, allowing us to obtain nutritious and healthy foods. Among the pulses, beans (*Phaseolus vulgaris* L.) are consumed worldwide and, moreover, with the growing of vegetarianism and the demand in Western countries for non-wheat and non-soy proteins, dry beans are obtaining increased attention by consumers; nevertheless, they are underutilized by the food industry as an ingredient in novel foods. Although, in general, all beans have a similar nutritive composition, each variety has a unique bioactive compound profile. In this study, the Almonga variety was used, which was previously reported to have a low lectin content, and whose consumption results in a significant decrease in triglyceride levels [7]. Considering that *Phaseolus vulgaris* lectin (PHA) is the main toxic compound present in beans, the use of this variety could be of great interest in the production of safe and healthy foods, notably, in the formulation of pasta products that require short cooking time. Some authors have developed rice-based pasta enriched with different pulses, although these pastas showed poor cooking quality, mainly due to a reduction in the integrity of the protein matrix [2,8].

A legume with high potential to be used as an ingredient to improve the quality of GF pasta is carob (*Ceratonia siliqua* L.). Carob seeds are utilized for the production of a thickener that is used in the food industry called carob bean or locus bean gum (E-410) [9,10]. Additionally, carob seeds contain a protein called caroubin, which has similar characteristics to wheat gluten but is safe for celiac people. Carob fruits are also rich in dietary fiber polyphenols and contain moderate amounts of inositol phosphates and α-galactosides [9,11]. The high amount of dietary fiber in carob fruit can weaken the firmness of the pasta, since it can disturb the protein matrix. Biernacka et al. enriched wheat pasta with up to 4% carob fiber with little effect on pasta quality and with good overall acceptability [12]. Therefore, the inclusion of a limited amount of whole carob fruit (WCF; pod and seeds) can be a promising ingredient in the development of good quality GF pasta and can obtain functional products [13] due to the presence of locus bean gum and caroubin in the seeds, as well as the presence of phenolic compounds in the pods. Some authors have reported the use of kibble, carob germ proteins, or carob bean gum in some bread or pasta products [12,14,15]. Turfani et al. [9] elaborated bread with lentil and carob seeds (up to 12%). In other previous works [4,11], the addition of WCF (5–10%) to extruded ’ready-to-eat‘ GF snacks based on rice/bean or rice/pea has created foods with a good bioactive compound content able to promote health functions and to improve the textural attributes of the snacks. Although, to the best of our knowledge, there are no studies regarding the development of novel pasta products containing both WCF plus another legume.

It is important to note that the content of bioactive compounds in any end-food product depends on the processing technique utilized in their development (such as dehulling, canning, soaking, germination, fermentation, autoclaving, extrusion-cooking, cold extrusion, etc.). Moreover, considering that pasta is consumed after cooking and that this process can induce changes in the phytochemical content, mainly in the heat-labile compounds [16,17], it is essential to know the real amount (i.e., that which would be consumed) present in the pasta after cooking. 

Taking into account the above information, the aim of this study was to determine the content of α-galactosides, mio-inositol phosphates, protease inhibitors, lectins, and phenols in dry (uncooked) and cooked experimental samples based on different proportions of rice/bean and supplemented with WCF, as well as in a commercial rice-based pasta whose phytochemical content has not been previously studied. The pasta quality was assessed by determining the texture and color properties. 

## 2. Materials and Methods

### 2.1. Raw Materials

White rice (*Oryza sativa* L.), raw beans (*P. vulgaris* var. Almonga), and whole carob fruit (WCF; pod and seeds) (*C. siliqua* L.) were the ingredients used to develop the GF pasta. The raw materials were acquired from Cámara Arrocera de Amposta (Tarragona, Spain), ITACyL (Instituto Tecnológico Agrario de Castilla y León, Valladolid, Spain), and Armengol Hermanos (Tarragona, Spain), respectively. All raw materials were milled and passed through a 1 mm sieve (Retsch SK1, Haan, Germany), and then stored in polyethylene bags until pasta elaboration. In order to compare the experimental pasta with a commercial product, a commercial fresh rice pasta was purchased from a local market.

### 2.2. Pasta Formulations and Manufacturing

Ten pasta formulations were elaborated by mixing different ratios of rice and bean, and supplemented with 10% whole carob bean (Table 1). The pasta was elaborated following the methodology (with minor modifications) of Gallegos-Infante et al. and Giuberti et al. [8,18]. The total amount of each formulation processed was 1 kg. To elaborate the fettuccine-shaped pasta, the different formulations were mixed (5 min) with an appropriate amount of hot water (46% on average) in a domestic blender (Thermomix TM-31, Vorweck, Wuppertal, Germany) to allow a uniform distribution of the water throughout the flour particles. The hydrated flours were worked in the mixing chamber of a continuous pilot-scale extruder for pasta production (Imperia & Monferrina S.p.A., Dolly, Moncalieri, Italy) for 15 min at constant speed to obtain aggregates of a 3–5 mm diameter. Afterward, a conventional cold (30–40 °C) extrusion was carried out in the same continuous pilot-scale extruder to produce experimental fettuccine (20 cm in length). The pasta was pre-dried at room temperature (30 min) and then in an oven at 70 °C (around 2 h). The commercial fresh rice pasta was dried in the same way as the experimental samples (Figure S1). Five hundred grams of each dried pasta was milled and passed through a 1 mm sieve, and then stored in polyethylene bags. The other 500 g of dried pasta was stored at room temperature in polyethylene boxes until required for the cooking procedure and the texture and color determinations. 

The dry pasta (P-) was elaborated and the commercial sample was cooked (PC-) at the optimal cooking time (OCT). The OCT was analyzed in a previous paper, and was 4 min for the P-Commercial rice, 2 min for P-Rice, and for the rest of the experimental fettuccine, it ranged from 2.6 min for P-20.0 to 3.92 min for P-80.10 [19]. All of the cooked samples were frozen immediately (−20 °C), lyophilized, and stored in polyethylene bags until required; then, they were ground in a mill equipped with a 1 mm sieve (Retsch SK1 mill, Haan, Germany).

### 2.3. Biochemical Characterization

All of the biochemical analyses were performed in the uncooked (P-) and the cooked samples (PC-).

#### 2.3.1. Individual Inositol Phosphates

The individual phosphates, from inositol triphosphate (IP3) to phytic acid (IP6), were determined according to the method of Burbano et al. [20]. Sodium phytate was used as the standard (Sigma-Aldrich, St. Louis, MO, USA). The samples were analyzed using high performance liquid chromatography (HPLC) (Beckman System Gold Instrument, Los Angeles, CA, USA) equipped with a refractive index detector and a macroporous polymer PRP-1 column (150 × 4.1 mm i.d., 5 µm, Hamilton, Reno, Nevada, USA) maintained at 45 °C with a flow rate of 1 mL/min. The mobile phase consisted of a mixture of methanol/water (52/48 *v*/*v*) plus 8 mL tetrabutylammonium hydroxide (40% in water), 1 mL 5 M sulphuric acid, 0.5 mL 91% formic acid, and 100 μL of phytic acid (6 mg/mL), and the pH was adjusted to 4.3.

#### 2.3.2. Soluble Sugars and α-Galactosides

The content of soluble sugars and α-galactosides was analyzed by HPLC (Beckman System Gold Instrument, Los Angeles, CA, USA) with a refractive index detector according to Pedrosa et al. [21]. The sugars present in each sample (0.1 g) were extracted with ethanol/water (50/50, *v*/*v*). Then, the extract was purified using Sep-Pack C18 cartridges (500 mg, Waters, Milford, MA, USA) and injected into the HPLC system. The mobile phase consisted of acetonitrile/water 60:40 (*v*/*v*) and was used in isocratic mode to equilibrate the column (Spherisorb-5-NH2, 250 × 4.66 mm i.d. Waters, Milford, MA, USA) at a 1 mL/min flow rate. The content of individual sugars was quantified by comparison to their corresponding standards (Sigma-Aldrich, St. Louis, MO, USA). Ciceritol was purified and kindly supplied by Dr. A. I. Piotrowicz-Cieslak (Olsztyn-Kortowo, Poland). 

#### 2.3.3. Trypsin and Chymotrypsin Inhibitors

The trypsin (TI) and chymotrypsin (CI) inhibitors were extracted according to Pedrosa et al. [21]. α-N-benzoyl-DL-arginine-p-nitroanilide hydrochloride (BAPNA) and benzoyl-L-tyrosine ethyl ester (BTEE) were used as substrate of the trypsin and the chymotrypsin, respectively. Trypsin inhibitor activity was determined at 410 nm, and one unit of trypsin inhibitor (TIU) per milligram of flour was defined as a decrease of 0.01 units of absorbance at 410 nm in relation to the trypsin control, using a 10 mL assay volume [22]. Chymotrypsin inhibitor activity was determined at 256 nm. One unit of chymotrypsin inhibitor (CIU) was stated as an increase of 0.01 absorbance units at 256 nm after 7 min after the addition of the substrate to the reaction mixture. 

#### 2.3.4. Lectin

The *Phaseolus vulgaris* lectin (PHA) content was determined using a non-commercial competitive indirect ELISA assay according to Cuadrado et al. [23]. The PHA content of the samples was calculated using a calibration curve (0.001–1000 μg/mL) of pure PHA standard. The limit of detection and of quantification were estimated from the regression equation calculated from the calibration curve (y = −0.4311Ln(x) + 2.711; r^2^ = 0.98). *P. vulgaris* cvs. Processor and Pinto were included in each assay as positive and negative controls, respectively, to determine the repeatability of the method. The results are expressed as percentage of PHA on a dry matter basis. 

#### 2.3.5. Phenolic Composition and Antioxidant Activity

A solution of methanol–HCl (1‰)/water (80:20 *v*/*v*) was used to extract the phenolic compounds of the different samples for 16 h at room temperature [24]. The different groups of phenols present in the extracts were quantified spectrophotometrically following the methodology of Oomah et al. [25]. Anthocyanins, flavonols, tartaric esters, and the total phenolic compounds were monitored at 520, 360, 320, and 280 nm, respectively, on a Beckman spectrofotometer (Beckman DU-640, Los Angeles, CA, USA). Commercial standards of cyanidin-3-glucoside from Extrasynthese (Germay, France), quercetin, caffeic acid, and catechin from Sigma-Aldrich (St. Louis, MO, USA) were used to quantify anthocyanins (μg of cyanidin-3-glucoside equivalents (C3GE) per gram dry weight), flavonols (μg of quercetin equivalents (QE) per gram of dry weight), tartaric esters (mg of caffeic acid equivalents (CAE) per gram of dry weight) and total phenols (mg of (+) catechin equivalents (CE) per gram of dry weight), respectively. The same extracts were used to determine the antioxidant activity by using the oxygen radical absorbance capacity (ORAC) assay [11]. A microplate fluorescence reader (FLUOstar Omega, BMG Labtech, Offenburg, Germany) was used with excitation and emission wavelengths at 485 and 530 nm, respectively. The ORAC values were calculated from a calibration curve of Trolox (0–8 μg/mL) and the results were expressed as μmol Trolox per gram of dry weight). 

### 2.4. Color Analysis

The color of the uncooked and the cooked fettuccine pastas in the optimum cooking time was measured using the Konica Minolta model CM-5 spectrophotometer (Konica, Minolta, Ramsey, NJ, USA) with a xenon lamp. The results are expressed in the color space CIE L*a*b*, where L* measures the degree of luminosity (lightness/darkness), a* (redness/greenness) where +a* are red tones and -a*are green tones, and b* (yellowness/blueness), where +b* yellow tones and -b* blue. The results are the mean of three replicates per sample, measured in each replica per quadruplicate [26]. The calibration (zero and white) was carried out (with zero calibration box and an internal white plate, respectively) and used to standardize daily the equipment before the measurements.

### 2.5. Texture Analysis

The evaluation of the texture [27] of the cooked (OCT) pasta was done using a texturometer (Model TA-XTplus, Stable Micro System, Surrey, UK) equipped with a load cell of 30 kg coupled to an aluminum P25 mm probe and calibrated daily using a 5 kg load cell. The instrument settings were as follows: compression mode with force pre-set, test, and post-test speed of 2 mm/s and deformation of 75%. The parameters measured were: hardness (force maximum positive), stickiness (force maximum negative), and adhesiveness (negative area). In addition, a cutting test was carried out with a Warner Bratzler at a speed of 0.17 mm/min, being the parameters determined: firmness (force maximum) and consistency (area up to the breaking point of the pasta). 

All of the measurements were carried out after cooling (1 min) the samples in cool water in order to end the cooking process. The results are expressed as the mean of four measurements from three different cooking replications of each sample. Texturometer software (Exponent Stable Micro System version 5.0.9.0; Stable Micro System, Surrey, UK) was used to record and calculate the values of the parameters analyzed.

### 2.6. Statistical Analysis

Results are presented as mean ± standard deviation (SD). They were obtained in quadruplicate, except for color and texture analysis (*n* = 12). The statistically significant differences (*p* < 0.05) were established by a one-way ANOVA, and a Duncan’s multiple range test was applied. Correlations were analyzed by Pearson’s test. In addition, data from the different chemical analyses were subjected to a principal component analysis (PCA), shown in the supplementary files (Figures S2 and S3). The Statgraphics Centurion XVI computer package (Graphics Software System, Rockville, MD, USA) was used.

## 3. Results and Discussion

The data corresponding to the different bioactive compounds present in the raw samples are shown in the corresponding tables (Table 2, Table 3, Table 4 and Table 5). Considering these results (on a dry basis), raw bean contained the highest amount of total inositol phosphate (IP), α-galactosides, and protease inhibitors, while WCF presented the highest content of sucrose and phenolic compounds. Raw bean revealed very low amounts of lectins, while rice presented low amounts of sucrose and trypsin inhibitors. The content of bioactive compounds determined in these raw materials was similar to that reported in the literature [11,12,16,28]. 

### 3.1. Individual Inositol Phosphates

The total and individual inositol phosphates (IP) content in the uncooked (P-) and cooked (PC-) fettuccine, as well as in the commercial rice pasta, are presented in Table 2.

As expected, a higher total IP content corresponded to the samples with a higher bean percentage. A linear correlation between the percentage of bean flour in the formulas and the total inositol phosphates content in uncooked and cooked samples was fitted according to the model: Total IP (mg/g) = 2.071 + 0.067 × %bean, and it showed a high correlation (R^2^ = 0.96). 

In the experimental dry or uncooked fettuccine, P-Bean showed the highest total IP content (9.03 mg/g), and P-Commercial rice pasta presented the lowest value (0.84 mg/g) (Table 2). On the other hand, the IP content in the cooked samples ranged between 1.16 and 7.97 mg/g for the PC-Commercial rice pasta and PC-Bean, respectively. There were no significant differences in the total IP content between the uncooked and cooked samples (except for the 80.10 formula), probably due to the heat-stable characteristics of phytic acid [29]. In general, the total inositol content showed a slight increase, which was not statistically significant, in the cooked samples (PC-). This could be due to the fact that during pasta-making, some inositol can form complexes with other food components (e.g., minerals, proteins, or starch), which would be released from the pasta matrix during cooking [30]. This slight increase could also be related to the losses of some components easily soluble in water, such as sugars or phenols (Table 3 and Table 5), that migrate to the cooking solution and that are discarded; this reduces the total dry matter of the samples and causes changes in the percentage of the specific components expressed on a dry matter basis [31]. The obtained results were in concordance with those published by Tazrart et al. [32] in maccheronccini elaborated with durum wheat semolina fortified with different ratios of faba bean flour (*Vicia faba* L.). In contrast, soaking yellow field peas (*Pisum sativum* L.) revealed that the levels of IP6 were reduced, and, also, the canning process of ‘ready-to-eat’ Spanish beans decreased significantly the total IP content [17].

These differences could be due to the differences in the cooking time and the temperature used to process the pulses. IP6 was the main form determined in all of the uncooked and cooked samples studied, accounting for, in general, about 65–75% of the total IP content, similar to the results published for different legumes by Burbano et al. [20]. In comparison to the commercial sample, the experimental fettuccine showed from 4 to 15 times more IP6 content, which is in concordance with the results published by Bilgicli et al. [33] for GF noodles fortified with legume (chickpea or soya). The same tendency was shown in semolina-based pasta enriched by α-galactosides-free lupin flours [34]. 

The amount of the less-phosphorylated IP forms (IP3–IP5) was similar in the uncooked and cooked samples. Therefore, the thermal process did not produce significant differences in the IP isoform content of most of the samples, except in the formulations with a high bean content and WCF. On the other hand, the IP3 content represented about 2.55–6.15% of the total IP content, being detected in all of the samples except in the uncooked and cooked rice 100% and the commercial rice samples (Table 2).

Although, phytic acid (IP6) can reduce the availability of some minerals and, at present, there is not a recommended dietary intake of phytate. Fredrikson et al. (2001) [35] reported that a content of total IP < 20 mg/g (as in the experimental fettuccine) allows a similar degree of mineral availability for commercial food based on soybean protein. Phytic acid has been linked to the epidemiological relation between the high-fiber diets (rich in IP6) and the low incidence of some cancers [11,16]. Also, it can reduce the toxicity of heavy metals (such as lead and cadmium) present in the diet. The less-phosphorylated isoforms (IP3–IP5) have been shown to be related to beneficial effects in human health, such as improving the hypercholesterolemia and the atherosclerosis, and preventing the formation of kidney stones, colon cancer, type 2 diabetes, and irritable bowel syndrome. The results obtained in the experimental fettuccine suggests that the cooking process was not strong enough to significantly affect the contents of phytic acid and the less-phosphorylated forms (IP5–IP3); consequently, they could retain the health benefits associated with the less-phosphorylated inositol phosphates. 

### 3.2. Soluble Sugars and α-Galactosides

The content of the different soluble sugars detected in the uncooked (P-) and cooked (PC-) fettuccine, as well as in the commercial rice pasta, are presented in Table 3. In the current study, sucrose was detected in all of the formulations, and significant differences (*p* < 0.05) between uncooked and cooked counterparts were observed. P-Rice was the sample with highest sucrose content (64.46 mg/g), whereas PC-20.0 showed the lowest amount (5.76 mg/g). As WCF had a high content of sucrose, the experimental fettuccine formulated with carob contained higher (*p* < 0.05) sucrose content than those formulations without this legume (e.g., P-60.0 vs. P-60.10 or PC-60.0 vs. PC-60.10). 

Raffinose, ciceritol, and stachyose were detected in all of the uncooked and cooked fettuccine samples, except in the formulation elaborated with 100% rice and the commercial sample. The main α-galactoside detected in the uncooked and cooked samples was stachyose, accounting for around 55–84% of the total α-galactosides, except in P-20.0 and P-20.10 (samples with a higher content of raffinose). The total α-galactosides content was significantly higher in both the uncooked and the cooked samples formulated with carob bean fruit. 

In general, the cooking process reduced (*p* < 0.05) the content of the individual sugars determined in each fettuccine sample. During the cooking process, pasta is hydrated and a softening of the food matrix occurs, facilitating the leaching of the sugars into the broth. The different sugars are not affected to the same extent by the cooking process, with the reduction of the individual sugars analyzed being from 4% (stachyose) to 88% (raffinose). This fact may be related to the different water solubility of these low-molecular weight sugars that migrate into the cooking water, which is discarded. These results are in accordance to those published by Laleg et al. [36] in gluten-free pasta elaborated exclusively from faba, lentil, or black-gram flours. The higher percentage of bean in the uncooked and cooked samples caused a higher α-galactosides content. Consequently, a good linear adjustment (R^2^ = 0.85) to the model was observed: total α-galactosides (mg/g) = 0.858 + 0.359 × %bean. The sugar profile of the uncooked and cooked fettuccine elaborated with 100% rice and the commercial rice pasta did not contain α-galactosides—they only presented sucrose and maltose (50% of each sugar, approximately). 

It should be noted that α-galactosides are traditionally associated with the production of flatulence, diarrhoea, and abdominal pain, since humans cannot digest these compounds, and they are used as substrates for anaerobic fermentation in the colon. Nowadays, α-galactosides are considered as nutritionally active factors or bioactive components with prebiotic activity by promoting the beneficial activity of intestinal microflora (bifidobacterias, bacteroides, and eubacteria), and they positively affect the immune system; thus, in humans they exert their health benefits through improving gut health, reducing constipation and diarrohea, stimulating the immune system, and increasing resistance to infection [16,37,38]. 

The commercial rice and the experimental fettuccine composed of 100% rice flour did not contain α-galactosides; thus, these samples did not have the health benefits associated with α-galactosides. The rest of the formulations showed high contents of α-galactosides and, as a consequence, they can exert health benefits associated with their consumption. There is not a dietary reference intake, although Martinez-Villaluenga et al. [39] reported that to obtain health benefits, an effective daily dose of α-galactosides is 3 g/day, since higher doses could be associated with the anti-nutritional problems mentioned above. Considering one serving of 60 g (dry weight) per day of fettuccine, a supply of between 0.67 g (PC-20.0) and 1.65 g (PC-80.0) of α-galactosides can be achieved. Thus, according to the above data [37], all of the experimental samples could promote health functions related to these compounds.

### 3.3. Trypsin and Chymotrypsin Inhibitors

Table 4 shows the trypsin and chymotrypsin inhibitor activities corresponding to the uncooked (P-) and cooked (PC-) fettuccine, as well as to the commercial rice pasta. The fettuccine (uncooked and cooked) elaborated with 100% rice and the commercial rice sample showed lower trypsin and chymotrypsin inhibitor activities, whereas P-Bean (13.19 TIU/mg and 16.87 CIU/mg) and PC-Bean (12.12 TIU/mg and 16.62 CIU/mg) were the samples with the highest protease inhibitor activities. The higher the bean content, the higher the inhibitor activities in all of the formulations; in fact, a strong linear correlation between the percentage of bean and protease inhibitor activities was fitted according to the models: TIU/mg = 0.454 + 0.122 × %bean (R^2^ = 0.98) and CIU/mg = 0.597 + 0.156 × %bean (R^2^ = 0.92). 

Thermal processing of legumes can be applied to reduce totally or partially the protease inhibitor activities due to the fact that they are thermo-labile compounds [17,39]. In general, the cooked fettuccine did not show significant (*p* > 0.05) changes in these activities compared to the uncooked samples. A slight increase (*p* > 0.05) was observed in both types of protease inhibitor activities after cooking, except in some samples: PC-80.0 and PC-Bean for trypsin inhibitor activity, and PC-20.10, PC-40.0, PC-60.0, and PC-60.10 for chymotrypsin inhibitor activity. This increase in their content (on a dry weight basis) would be due to the cooking losses, as has been reported above for other compounds [40,41]. 

The slight changes observed after cooking could also be due to the short cooking time applied (on average 3 min), and to the different heat sensitivity of both types of protease inhibitors. 

The results obtained in the experimental fettuccine are in concordance with the results of the trypsin inhibitor activity obtained by Zhao et al. [42] for uncooked and cooked wheat semolina spaghetti fortified with 20% of chickpea. On the contrary, Frias et al. [43] reported a reduction of trypsin inhibitor activity by around 52–59% after boiling macaroni elaborated with 100% green pea or yellow pea flours for 30 min. Even though pulse protease inhibitors interfere with protein digestibility [16], they are considered key players in several biological processes, including cancer progression, and they are associated with the capacity to prevent certain tumoral pathologies [5,42]. Thus, diets containing these compounds have been associated with a reduction in carcinogen-induced effects [41].

### 3.4. Lectin Content

Raw bean var. Almonga had a very low amount of lectin (Table 4), lower than the Pinto bean cultivar used as a negative (non-toxic) control; thus, their inclusion in foods that need a low cooking time can be of great interest to avoid the negative effect associated with raw or slightly cooked beans. The content of lectins in the different uncooked and cooked fettuccine evaluated by indirect ELISA is shown in Table 4. The results of ELISA show that the P-Bean sample presented the highest value of PHA (0.58%), while in the P-Rice and in P-Commercial rice samples, PHA was not detected. The uncooked fettuccine showed higher PHA values as the bean percentage in the samples increased, ranging from 0.26% to 0.58% (P-20.0 and P-Bean, respectively). 

The addition of carob bean to the formulations did not significantly increase the PHA content (e.g., P-40.0 vs. P-40.10). After the cooking process, there was a total reduction of the lectin content in all samples analyzed, in agreement with previous works [11,17] that indicated that the heat processing of different formulations containing bean or lentil was effective to eliminate their lectins. Taking into account that PHA is considered a toxic lectin, their absence after cooking made the experimental fettuccine a safe food, suitable for consumption without the risks to health (vomiting, diarrhoea, or bloating) that appear after consumption of raw or uncooked samples with high lectin content. 

### 3.5. Phenolic Composition and Antioxidant Activity

Table 5 presents the content of anthocyanins, flavonols, tartaric acid, and total phenols and their antioxidant activity (ORAC) determined in the uncooked (P-) and cooked (PC-) fettuccine and commercial samples. As it has been observed above for other bioactive compounds, the increase in the amount of bean in the formulation produced a rise in the total phenols content. Moreover, it is important to note that the supplementation of the different rice/bean samples with 10% WCF increases (*p* < 0.05) the content of phenolic compounds, both in the uncooked and the cooked samples. 

The uncooked fettuccine (P-) showed, in general, higher total phenols content (*p* < 0.05) than the cooked samples (PC-), except the fettuccine elaborated with 100% rice that did not show significant differences after cooking. These results are in concordance with the data of the raw materials (Table 5), since WCF and bean were the source of polyphenols in the elaborated fettuccine. Regarding the present study, the losses of the total phenols content after cooking the fettuccine (around 17–48%) were higher than that reported by Pedrosa et al. [16] after the canning process of Almonga bean seeds (by about 11%), but were similar to the results found in the literature in cooked pasta fortified with buckwheat, common and black beans, and red and black lentil pasta [18,43,44]. According to the literature [18,43,44,45], the reduction in phenols can be attributed to the synergistic combination of several factors, such as the pasta-making process, the sensitivity of heat during the cooking process, the leaching into the broth because of the softening of the food matrix that occurs during pasta cooking, as well as the association with the food matrix during cooking, that might hinder their extraction. Verardo et al. [46] reported for cooked buckwheat spaghetti that 11.6% of the different phenolic compounds were dissolved in the cooking water, and the pasta-making process caused a loss of 45.9% of the total phenolic compounds present in the raw materials used in the elaboration of buckwheat spaghetti.

Compared to the cooked commercial rice pasta, the cooked fettuccine fortified with WCF (10%) contained from 2.5-fold (PC-20.10) to 3.2-fold (PC-80.10) more phenols than the commercial sample, similar to other durum wheat pasta enriched with 1–5% carob pods [12].

Tartaric ester, flavonol, and anthocyanin content of the experimental fettuccine was also affected by the cooking process; the tartaric esters were reduced by around 7–50% and the flavonols between 10% and 75%, while the commercial rice sample did not reduce significantly their content after the cooking process. The anthocyanins were reduced (*p* < 0.05) in all the samples by the cooking process. The reduction ranged from 52% to 82% in the fettuccine without WCF (PC-20.0 and PC-60.0) and from 68% to 86% in the fettuccine with WCF (PC-20.10 and PC-60.10). The decrease was small in the samples with an 80% bean amount, where a reduction of 7% was observed; although the cause of this small reduction remains uncertain, a possible hypothesis is that in these samples, during the pasta-making and cooking processes, a higher amount of phenols are able to bind to the food matrix, which prevents its extraction [44].

In relation to the antioxidant activity (ORAC assay) (Table 5), the results show that the highest value corresponded to the uncooked sample P-80.10 (13.68 µmol Trolox/g), and the lowest corresponded to the P-Commercial Rice (4.38 µmol Trolox/g). The antioxidant activity was linked to the presence of phenols supplied by the bean and WCF flours, and to the presence of other antioxidant compounds such as the Maillard reaction products formed during the pasta-making drying step. As mentioned above for the phenolic compounds, the presence of WCF increased the antioxidant activity in comparison to the same formulations without this legume. After cooking, a slight drop (*p* > 0.05) of the antioxidant activity was observed; the ORAC values ranged from 3.08 to 10.32 µmol Trolox/g for the PC-Commercial rice and PC-60.10 samples, respectively. A similar decrease was reported by Rocchetti et al. [47] in six commercial GF pastas elaborated with black rice, chickpea, red lentil, sorghum, amaranth, and quinoa. The correlation between total phenols, tartaric esters, flavonols, and ORAC values was high, with a Pearson’s coefficient R^2^ of 0.88, 0.84, and 0.73, respectively (Table S1). 

It is well known that phenols have antioxidant, anti-inflammatory, and antimicrobial properties [48]. Consequently, they are preventive agents against several degenerative diseases, and in vitro, they are associated with a low incidence of several types of cancer (breast, skin, prostate, and colon cancer), as well as cardiovascular diseases [41]. Carob flour was described by Avallone et al. [14] as a rich source of phenolic compounds linked to antioxidant and cytotoxic activities. The daily phenols intake depends on specific dietary preferences and socio-cultural factors of different countries. In general, it is accepted that the total polyphenol intake for the overall population is about 900 mg/day [48,49]. At present, there is no a dietary recommended intake of phenolic compounds to exert these healthy effects; although, some authors have reported a minimum dose of 300 mg of total phenols to obtain health benefits. In this regard, one serving (60 g) of the experimental rice/bean fettuccine supplemented with 10% WCF could supply, on average, 300 mg of total phenols, that, in addition, represent a third of the daily intake of phenols. Thus, the consumption of the experimental rice/bean-based fettuccine supplemented with 10% WCF would be valuable for a balanced diet with some health-related functions. 

A principal component analysis (PCA) was carried out on the results obtained regarding the different bioactive compounds and the antioxidant activity (ORAC) of the uncooked (P-) and cooked (PC-) experimental fettuccine and the commercial rice pasta (control). The first three principal components explained 84.29% of the total variance (supplementary PCA file, Figures S2 and S3). The first two principal components (Figure S2) explained a relative percentage of 72.56%. PC1 was most correlated with total IP, total α-galactosides, tartaric esters, and antioxidant activity, and PC2 was most correlated with protease inhibitors and flavonols. The percentage of legumes in the experimental fettuccine was well-described by PC1, and the cooking effect was well–described by both PC1 and PC2. Lectin and anthocyanin content contributed, to a higher extent, to explaining the variance of PC3 (11.74%). 

### 3.6. Color Analysis

Color is a primary parameter for consumers’ acceptance of a product such as dry and cooked pasta. Food color without additives mainly depends on the composition of raw materials (protein, dietary fiber, phenols, etc.) used in the elaboration of the pasta [15]. In general, it was observed that an enrichment of wheat pasta samples with other kinds of flours caused an overall darkening of the pasta color shade, yielding a significant decrease in L* value [50]. 

The color values of the uncooked and cooked fettuccine, as well as those of the commercial sample, are shown in Table 6. The increase in bean percentage in the formulation did not cause significant changes in the luminosity (L*) of the uncooked fettuccine; however, there was an increase (*p* < 0.05) in the yellow hue (b*), similarly to that reported by Gallegos-Infante et al. [18]. The color parameters of both the cooked and uncooked fettuccine showed significant differences (*p* < 0.05) between the samples with or without WCF. The fettuccine supplemented with 10% WCF had lower values of L*, and they resulted in redder (a*) (*p* < 0.05) and less yellowish samples in comparison to the formulations with equal bean content but without WCF. These differences can be linked to the brown color of the raw WCF and the processing conditions, both in the making and the cooking processes [1,15]. 

These changes in the luminosity were strongly and positively correlated to the fortification with 10% WCF: L* = 69.563 − 1.902 × %WCF (R^2^ = −0.72); which is in concordance with the fortified pasta elaborated with 5% carob fiber reported by Chillo et al. [1]. The cooked fettuccine without WCF resulted in a less-red and less-yellow pasta than the cooked samples with WCF. In general, the cooked pasta was darker with brown hues, probably due to the transformation of carbohydrates by the reactions of Maillard during thermal processing [43]. The dry (P-) and cooked (PC-) commercial pasta had the highest lightness values (92.01 and 70.08, respectively) of all of the analyzed samples, while the fettuccine elaborated with 100% bean were the most yellow ones (22.85 and 19.98, P-Bean and PC-Bean, respectively). In the market, many types of colored pastas are available (whole grain, fortified with vegetables, inks, etc.), Thus, although the color of the novel pasta is different from the control pasta (Figure S1), they would be easily accepted by consumers. 

### 3.7. Texture Analysis

Regarding the textural parameters of cooked pastas, firmness and stickiness play an important role in the acceptability of pasta by consumers, where a sticky pasta is generally unacceptable [46]. The texture values (Table 7) of the GF pasta elaborated with rice and bean show an increase in the hardness as the percentage of bean increased in the formulation, corresponding to the highest hardness of 70.36 N in the PC-Bean fettuccine, as per the occurrence in precooked rice–yellow pea pasta and faba bean pasta reported by Bouasla et al. and Rosa-Sibakov et al. [50,51], respectively. Nevertheless, the supplementation of fettuccine with 10% WCF decreased the hardness compared to the same pasta without WCF, probably due to the increase in dietary fiber content, which may have led to the formation of crashes or breaks inside the fettuccine strand, thus weakening the pasta structure [46,52]. The adhesiveness of fettuccine, in general, showed a slight increase with the increase in the bean percentage; on the contrary, a higher percentage of bean produced lower stickiness. Bouasla et al. [51] reported the same tendency in precooked rice–yellow pea pasta. 

The addition of 10% WCF in the formulations produced, in general, a decrease in both the adhesiveness and the stickiness, although the changes in the adhesiveness were non-significant. The cooked samples with higher stickiness corresponded to the PC-20.0 and PC-Rice samples (9.21 N and 9.11 N, respectively). A reduction of the stickiness was also reported by other authors in amaranthus whole meal flour-based pasta fortified with quinoa, broad bean, or chickpea flour [1]. Both parameters are related to the amount of starch granules in the surface of the cooked pasta, the amount and type of dietary fiber, and the protein content. The manufacturing process utilized can also affect the textural parameters [2]. During the elaboration of the pasta by cold extrusion, the screw produced a mechanical heat able to pre-gelatinize some amount of the rice starch [53]. This pre-gelatinized starch, together with the unmodified starch granules, the high amount of fiber and protein, as well as the presence of caroubin (with similar properties of gluten), is responsible of the reduction in stickiness after cooking. 

Considering the values obtained regarding the firmness and consistency from the cutting analysis, it can be observed that an increase in the bean content produced an increase in the value of both parameters. These increases were significant for samples with more than a 60% bean content, with the corresponding maximum values belonging to the PC-Bean sample (4.7 N and 10.58 N·s, respectively), while the minimum values corresponded to the fettuccine elaborated with 100% rice (1.96 N and 3.53 N·s, respectively). The addition of WCF produced a decrease in both parameters, related to the weakening of the fettuccine matrix due to the increase in the dietary fiber and the decrease of starch content of the fettuccine [15], which is in concordance with the hardness values obtained above. 

In general, the elaborated fettuccine showed higher values of hardness (1.1–2 times) and stickiness (1.6–3.3 times), similar adherence, higher values of firmness (1.1–1.9 times), and lower consistency values (1.1–2.5 times) than the commercial sample analyzed. A firm and hard texture would be desirable because it allows a correct hydration of the pasta, which restricts the swelling of starch granules and prevents the easy fracturability that characterizes gluten-free pasta [36,54].

The changes in the texture parameters could also be related to the presence of bioactive compounds in the fettuccine. Taherian et al. [55] and Tsai et al. [56] reported that phytic acid (IP6) and phenols, respectively, can induce changes in the hardness of food products. To estimate the possible influence of bioactive compounds on the texture parameters, correlation Pearson’s coefficients were calculated. The results of the hardness and firmness parameters correlated well with most of the bioactive compounds analyzed (Table S2). In the case of hardness, a strong positive correlation was observed with total inositol phosphates (R^2^ = 0.84; *p* = 0.0000), total galactosides (R^2^ = 0.73; *p* = 0.0000), and protease inhibitors (R^2^ = 0.81; *p* = 0.0000). The firmness showed a moderate and positive correlation with the same bioactive compounds, with Pearson’s coefficients of 0.58 (*p* = 0.0000), 0.57 (*p* = 0.0000), and 0.63 (*p* = 0.0000) for inositol phosphates, galactosides, and protease inhibitors, respectively. 

## 4. Conclusions

The experimental fettuccine revealed a great health potential due to their bioactive compound composition. The inositol phosphates and protease inhibitors showed a slight increase, whereas α-galactosides, total phenols content, and antioxidant activity showed a decrease after cooking. Even though there is not a recommended dietary intake, according to the data found in the literature, the amount detected of these bioactive compounds in the different fettuccine would be enough to maintain their healthy characteristics (e.g., prebiotics, antioxidants, anticarcinogenic, etc.) and to reduce their associated drawbacks (e.g., flatulence, reduction of mineral bioavailability, impairment of digestion, etc.). The lectin content was eliminated by the cooking process, avoiding the toxic problems associated with their consumption. The addition of high percentages of bean improved the bioactive compound content, as well as the texture of the fettuccine. The pasta supplemented with 10% whole carob fruit was darker with brown hues, and showed better texture parameters (less hard, less sticky, and less adhesive) than the formulations without this legume. The improvement of the texture parameters observed could meet the expectation of consumers of GF products.

In comparison to the commercial sample, except that of 100% rice, all of the experimental fettuccine contained higher amounts of bioactive compounds, notably those supplemented with carob fruit, being a suitable nutritional and healthy alternative to the commercially available gluten-free rice-based pasta for celiac individuals and for the general population that consume gluten-free products.

## Figures and Tables

**Table 1:** Codification and formulation of the different rice/bean-based fettuccine pastas fortified with carob fruit flours and the commercial control pasta (external control).

Formulation	Rice (%)	Bean (%)	Carob Fruit (%)
20.0	80	20	0
20.10	70	20	10
40.0	60	40	0
40.10	50	40	10
60.0	40	60	0
60.10	30	60	10
80.0	20	80	0
80.10	10	80	10
Bean 100%	0	100	0
Rice 100%	100	0	0
Commercial rice	The product label: rice flour, corn flour, thickener and emulsifier additives, and water.		

Note: All of the experimental samples contained 1% of sodium chloride in the formulation.

**Table 2:** Inositol phosphates content (mg/g dry weight) of the raw materials and the different uncooked (P-) and cooked (PC-) fettuccine pastas and the commercial rice pasta.

Sample	IP3	IP4	IP5	IP6	Total Inositol Phosphates
**Bean**	0.26 ± 0.01	0.42 ± 0.01	1.39 ± 0.03	10.12 ± 0.03	12.20
**Carob fruit**	n.d.	0.15 ± 0.01	0.36 ± 0.04	0.15 ± 0.01	0.66
**Rice**	0.10 ± 0.01	0.03 ± 0.03	0.22 ± 0.01	1.53 ± 0.05	1.88
**P-20.0**	0.23 ± 0.001 ^e f, A^	0.39 ± 0.02 ^b, A^	0.71 ± 0.07 ^b, A^	2.51 ± 0.13 ^c, A^	3.83 ^c, A^
**P-20.10**	0.22 ± 0.001 ^c d, A^	0.36 ± 0.02 ^b, A^	0.70 ± 0.04 ^b, A^	2.51 ± 0.18 ^c, A^	3.79 ^c, A^
**P-40.0**	0.25 ± 0.02 ^j, A^	0.55 ± 0.03 ^d e A^	1.08 ± 0.04 ^c d, A^	3.29 ± 0.21 ^e f, A^	5.16 ^d, A^
**P-40.10**	0.22 ± 0.001 ^d e, A^	0.52 ± 0.05 ^c d, A^	0.97 ± 0.18 ^c, A^	3.11 ± 0.12 ^e, A^	4.83 ^d, A^
**P-60.0**	0.24 ± 0.01 ^h i, A^	0.56 ± 0.02 ^c d, A^	1.28 ± 0.07 ^d e, A^	3.57 ± 0.11 ^f g, A^	5.65 ^e, A^
**P-60.10**	0.24 ± 0.01 ^g h i, A^	0.64 ± 0.10 ^f, A^	1.18 ± 0.57 ^c d e, A^	4.09 ± 0.34 ^h, A^	6.15 ^f, A^
**P-80.0**	0.23 ± 0.01 ^f g, A^	0.66 ± 0.03 ^f g, A^	1.84 ± 0.08 ^f g, A^	4.80 ± 0.11 ^j, A^	7.53 ^h, A^
**P-80.10**	0.24 ± 0.01 ^i j, A^	0.70 ± 0.01 ^g, A^	1.97 ± 0.09 ^g, A^	5.16 ± 0.20 ^j, A^	8.06 ^i, A^
**P-Bean**	0.25 ± 0.001 ^h, A^	0.75 ± 0.02 ^h, A^	2.29 ± 0.12 ^h, A^	5.73 ± 0.58 ^k, A^	9.03 ^j, A^
**P-Rice**	n.d.	0.27 ± 0.02 ^a, A^	0.47 ± 0.12 ^a, A^	1.48 ± 0.13 ^b, A^	2.22 ^b, A^
**P-Commercial rice**	n.d.	0.25 ± 0.01 ^a, A^	0.29 ± 0.01 ^a, A^	0.37 ± 0.06 ^a, A^	0.84 ^a, A^
**PC-20.0**	0.22 ± 0.001 ^b c d, B^	0.39 ± 0.02 ^b, A^	0.74 ± 0.03 ^b, A^	2.81 ± 0.20 ^d, B^	4.15 ^c, A^
**PC-20.10**	0.21 ± 0.001 ^b, B^	0.38 ± 0.01 ^b, A^	0.76 ± 0.02 ^b, A^	2.40 ± 0.18 ^c, A^	3.75 ^c, A^
**PC-40.0**	0.22 ± 0.001 ^c d, B^	0.48 ± 0.02 ^c, B^	1.07 ± 0.07 ^c d, A^	3.35 ± 0.08 ^e f, A^	5.12 ^d, A^
**PC-40.10**	0.21 ± 0.001 ^b c d, A^	0.48 ± 0.03 ^c, B^	1.18 ± 0.11 ^c d e, A^	3.35 ± 0.06 ^e f, A^	5.23 ^d, A^
**PC-60.0**	0.23 ± 0.001 ^e f, B^	0.52 ± 0.03 ^c d e, A^	1.27 ± 0.10 ^d e, A^	3.84 ± 0.12 ^g h, A^	5.86 ^e f, A^
**PC-60.10**	0.22 ± 0.001 ^d e, B^	0.54 ± 0.03 ^d e, B^	1.39 ± 0.06 ^e, A^	3.91 ± 0.17 ^h, A^	6.05 ^f, A^
**PC-80.0**	0.23 ± 0.001 ^f g h, A^	0.63 ± 0.04 ^f, A^	1.76 ± 0.12 ^f g, A^	4.82 ± 0.16 ^j, A^	7.43 ^h, A^
**PC-80.10**	0.23 ± 0.001 ^f g, B^	0.57 ± 0.01 ^e, B^	1.64 ± 0.05 ^f, B^	4.54 ± 0.32 ^j, B^	6.98 ^g, B^
**PC-Bean**	0.21 ± 0.001 ^b c, B^	0.57 ± 0.03 ^c d, A^	1.91 ± 0.08 ^g, B^	5.28 ± 0.11 ^j, B^	7.97 ^i, A^
**PC-Rice**	n.d.	0.26 ± 0.01 ^a, A^	0.46 ± 0.03 ^a, A^	1.40 ± 0.08 ^b, A^	2.12 ^b, A^
**PC-Commercial rice**	n.d.	0.29 ± 0.01 ^a, A^	0.34 ± 0.03 ^a, A^	0.53 ± 0.12 ^a, A^	1.16 ^a, A^

Values are means ± standard deviation (*n* = 4). Mean values in the same column followed by a different superscript letter are significantly different (*p* < 0.05); small superscript letters mean differences among all of the samples analyzed, whereas capital superscript letters mean differences due to the cooking treatment for the same formulation. n.d., not detected.

**Table 3:** Content of soluble sugars, ciceritol, and α-galactosides (mg/g dry weight) in raw materials and in the uncooked (P-) and cooked (PC-) fettuccine, as well as in the commercial rice pasta.

Sample	Sucrose	Maltose	Raffinose	Ciceritol	Stachyose	Total α-Galactosides
**Bean**	30.00 ± 0.95	n.d.	5.92 ± 0.09	0.34 ± 0.01	26.85 ± 0.25	32.77
**Carob fruit**	150.46 ± 10.04	n.d.	5.84 ± 0.02	n.d.	n.d.	5.84
**Rice**	2.98 ± 0.15	n.d.	n.d.	n.d.	n.d.	n.d.
**P-20.0**	8.59 ± 0.39 ^b, A^	n.d.	6.40 ± 0.58 ^e, A^	2.73 ± 0.30 ^b, A^	3.95 ± 0.38 ^a, A^	10.35 ^d, A^
**P-20.10**	25.46 ± 0.09 ^I, A^	n.d.	8.31 ± 0.16 ^g, A^	2.19 ± 0.22 ^b, A^	3.47 ± 0.25 ^a, A^	11.78 ^e, A^
**P-40.0**	14.18 ± 0.34 ^f, A^	n.d.	9.11 ± 0.27 ^h, A^	4.71 ± 0.08 ^e, A^	9.06 ± 0.18 ^b, A^	18.17 ^h, A^
**P-40.10**	36.70 ± 0.78 ^p, A^	n.d.	8.00 ± 0.80 ^g, A^	6.43 ± 0.14 ^f, A^	13.37 ± 0.32 ^d, A^	21.37 ^j, A^
**P-60.0**	23.77 ± 0.74 ^k, A^	n.d.	9.05 ± 0.29 ^h, A^	7.88 ± 0.17 ^g, A^	17.25 ± 0.37 ^f, A^	26.30 ^k, A^
**P-60.10**	41.98 ± 0.68 ^q, A^	n.d.	13.13 ± 0.22 ^j, A^	10.09 ± 0.27 ^h, A^	17.41 ± 0.36 ^f, A^	30.54 ^m, A^
**P-80.0**	29.63 ± 1.06 ^n, A^	n.d.	11.84 ± 0.89 ^i, A^	12.90 ± 0.94 ^j, A^	26.92 ± 1.45 ^i, A^	38.76 ^n, A^
**P-80.10**	57.44 ± 0.52 ^r, A^	n.d.	18.72 ± 0.20 ^l, A^	13.55 ± 0.48 ^k, A^	26.12 ± 0.41 ^h, A^	44.85 ^o, A^
**P-Bean**	36.53 ± 0.93 ^p, A^	n.d.	13.82 ± 0.25 ^k, A^	12.36 ± 0.18 ^i, A^	30.71 ± 0.34 ^j, A^	44.53 ^o, A^
**P-Rice**	64.46 ± 1.44 ^s, A^	64.77 ± 1.30 ^d, A^	n.d.	n.d.	n.d.	n.d.
**P-Commercial rice**	18.32 ± 0.35 ^h, A^	15.51 ± 1.21 ^b, A^	n.d.	n.d.	n.d.	n.d.
**PC-20.0**	5.76 ± 0.44 ^a, B^	n.d.	0.77 ± 0.08 ^a, B^	1.37 ± 0.17 ^a, B^	3.73 ± 0.06 ^a, A^	4.51 ^b, B^
**PC-20.10**	18.00 ± 0.28 ^g, B^	n.d.	1.77 ± 0.09 ^b, B^	2.54 ± 0.17 ^b, A^	3.89 ± 0.12 ^a, A^	5.67 ^c, B^
**PC-40.0**	9.66 ± 0.35 ^c, B^	n.d.	1.86 ± 0.23 ^b, B^	4.06 ± 0.28 ^d, B^	9.78 ± 0.18 ^b, A^	11.64 ^e, B^
**PC-40.10**	22.37 ± 0.54 ^j, B^	n.d.	4.03 ± 0.31 ^b, B^	4.82 ± 0.79 ^e, B^	11.17 ± 1.02 ^c, B^	15.20 ^g, B^
**PC-60.0**	11.76 ± 0.16 ^d, B^	n.d.	2.14 ± 0.11 ^c, B^	3.41 ± 0.14 ^c, B^	11.01 ± 0.09 ^c, B^	13.15 ^f, B^
**PC-60.10**	27.20 ± 1.81 ^m, B^	n.d.	5.13 ± 0.23 ^d, B^	3.52 ± 0.23 ^c, B^	10.74 ± 0.50 ^c, A^	15.87 ^g, B^
**PC-80.0**	21.02 ± 0.55 ^i, B^	n.d.	7.03 ± 0.05 ^f, B^	7.66 ± 0.03 ^g, B^	20.51 ± 0.72 ^g, B^	27.54 ^l, B^
**PC-80.10**	30.28 ± 0.81 ^n, B^	n.d.	7.33 ± 0.12 ^f, B^	4.70 ± 0.20 ^e, B^	19.34 ± 0.23 ^e, B^	26.67 ^l, B^
**PC-Bean**	17.57 ± 0.41 ^g, B^	n.d.	3.72 ± 0.29 ^c, B^	3.31 ± 0.12 ^c, B^	15.89 ± 0.35 ^e, B^	19.61 ^i, B^
**PC-Rice**	12.80 ± 0.11 ^e, B^	10.85 ± 0.71 ^a, B^	n.d.	n.d.	n.d.	n.d.
**PC-Commercial rice**	34.55 ± 0.33 ^o, B^	24.06 ± 0.79 ^c, B^	n.d.	n.d.	n.d.	n.d.

Values are means ± standard deviation (*n* = 4). Mean values in the same column followed by a different superscript letter are significantly different (*p* < 0.05); small superscript letters mean differences among all of the samples analyzed, whereas capital superscript letters mean differences due to the cooking treatment for the same formulation. n.d., not detected.

**Table 4:** Content of trypsin inhibitors (TIU/mg dry weight), chymotrypsin inhibitors (CIU/mg dry weight), and lectin content (%PHA) in the uncooked (P-) and cooked (PC-) fettuccine samples and in the commercial rice pasta.

Sample	Trypsin Inhibitors	Chymotrypsin Inhibitors	Lectin
**Bean**	23.21 ± 0.66	7.74 ± 0.28	0.59 ± 0.01
**Carob fruit**	0.30 ± 0.02	n.d.	-
**Rice**	0.15 ± 0.01	n.d.	-
**P-20.0**	2.33 ± 0.07 ^b, A^	2.98 ± 0.25 ^b c, A^	0.26 ± 0.04 ^a^
**P-20.10**	3.10 ± 0.28 ^c, A^	2.76 ± 0.23 ^b, A^	0.36 ± 0.17 ^a b^
**P-40.0**	5.67 ± 0.08 ^d, A^	5.85 ± 0.54 ^d, A^	0.38 ± 0.10 ^a b^
**P-40.10**	6.19 ± 0.40 ^d e, A^	8.22 ± 0.84 ^g, A^	0.42 ± 0.12 ^a b^
**P-60.0**	7.57 ± 0.56 ^f, A^	10.85 ± 1.05 ^h, A^	0.34 ± 0.18 ^a b^
**P-60.10**	7.25 ± 0.51 ^f, A^	10.23 ± 1.08 ^h, A^	0.39 ± 0.12 ^a b^
**P-80.0**	10.26 ± 0.77 ^g, A^	16.15 ± 0.75 ^j, A^	0.54 ± 0.10 ^b^
**P-80.10**	10.19 ± 0.42 ^g, A^	7.70 ± 0.32 ^e f, A^	0.56 ± 0.28 ^b^
**P-Bean**	13.19 ± 0.75 ^j, A^	16.87 ± 0.57 ^j, A^	0.58 ± 0.14 ^b^
**P-Rice**	0.50 ± 0.04 ^a, A^	0.56 ± 0.13 ^a, A^	n.d.
**P-Commercial rice**	0.66 ± 0.03 ^a, A^	0.40 ± 0.10 ^a, A^	n.d.
**PC-20.0**	2.45 ± 0.07 ^b, A^	3.41 ± 0.34 ^b c, A^	n.d.
**PC-20.10**	3.17 ± 0.22 ^c, A^	4.01 ± 0.86 ^c, B^	n.d.
**PC-40.0**	5.67 ± 0.35 ^d, A^	6.85 ± 0.61 ^e, B^	n.d.
**PC-40.10**	6.35 ± 0.36 ^e, A^	7.45 ± 0.74 ^e f, A^	n.d.
**PC-60.0**	6.96 ± 0.37 ^f, A^	12.26 ± 1.01 ^i, B^	n.d.
**PC-60.10**	7.13 ± 0.72 ^f, A^	12.08 ± 1.55 ^i, B^	n.d.
**PC-80.0**	11.10 ± 0.34 ^h, B^	16.30 ± 1.27 ^j, A^	n.d.
**PC-80.10**	10.24 ± 0.74 ^g, A^	8.21 ± 0.72 ^g, A^	n.d.
**PC-Bean**	12.12 ± 0.35 ^i, B^	16.62 ± 1.10 ^j, A^	n.d.
**PC-Rice**	0.15 ± 0.02 ^a, A^	0.47 ± 0.01 ^a, A^	n.d.
**PC-Commercial rice**	0.33 ± 0.01 ^a, A^	0.29 ± 0.02 ^a, A^	n.d.

Values are means ± standard deviation (*n* = 4). Mean values in the same column followed by a different superscript letter are significantly different (*p* < 0.05); small superscript letters mean differences among all of the samples analyzed, whereas capital superscript letters mean differences due to the cooking treatment for the same formulation. n.d., not detected.

**Table 5:** Anthocyanins (μg C3GE/g dry weight), flavonols (μg QE/g dry weight), tartaric esters (mg CAE/g dry weight) and total phenols (mg (+) CE/g dry weight) content and the antioxidant activity (µmol Trolox/g dry weight) of the raw materials and the uncooked (P-) and cooked (PC-) fettuccine, as well as the commercial rice pasta.

Sample	Total Phenols	Tartaric Esters	Flavonols	Anthocyanins	Antioxidant Activity (ORAC)
**Bean**	2.88 ± 0.02	0.21 ± 0.01	0.08 ± 0.001	36.96 ± 0.24	24.33 ± 0.07
**Carob fruit**	20.73 ± 0.10	0.72 ± 0.01	0.75 ± 0.001	18.00 ± 0.15	69.89 ± 1.62
**Rice**	0.90 ± 0.03	0.02 ± 0.001	0.03 ± 0.001	18.70 ± 0.83	3.80 ± 0.30
**P-20.0**	2.88 ± 0.20 ^d e, A^	0.07 ± 0.001 ^c d, A^	0.03 ± 0.001 ^c, A^	10.65 ± 0.001 ^g h, A^	5.26 ± 0.65 ^c d, A^
**P-20.10**	5.77 ± 0.06 ^l, A^	0.14 ± 0.02 ^i, A^	0.10 ± 0.001 ^g, A^	10.64 ± 0.001 ^g h, A^	10.49 ± 0.69 ^g h, A^
**P-40.0**	4.58 ± 0.01 ^g h, A^	0.09 ± 0.001 ^e f, A^	0.03 ± 0.001 ^b c, A^	8.25 ± 0.001 ^e f g h, A^	6.94 ± 0.67 ^f, A^
**P-40.10**	5.40 ± 0.21 ^j k, A^	0.14 ± 0.001 ^i, A^	0.08 ± 0.001 ^f, A^	9.16 ± 0.001 ^f g h, A^	9.67 ± 1.23 ^h i, A^
**P-60.0**	4.70 ± 0.40 ^g h i, A^	0.09 ± 0.001 ^f, A^	0.03 ± 0.001 ^c, A^	8.25 ± 0.001 ^d e f g h, A^	9.20 ± 0.52 ^g h, A^
**P-60.10**	5.80 ± 0.31 ^k l, A^	0.15 ± 0.001 ^j, A^	0.10 ± 0.001 ^g, A^	6.32 ± 0.001 ^b c d e f, A^	12.39 ± 0.27 ^k, A^
**P-80.0**	4.48 ± 0.34 ^g, A^	0.12 ± 0.001 ^g, A^	0.04 ± 0.001 ^d, A^	5.70 ± 0.001 ^b c d e f, A^	11.06 ± 0.64 ^j, A^
**P-80.10**	7.27 ± 0.21 ^m, A^	0.20 ± 0.01 ^k, A^	0.10 ± 0.001 ^g, A^	6.87 ± 0.001 ^c d e f g, A^	13.68 ± 0.58 ^l, A^
**P-Bean**	5.11 ± 0.13 ^i j, A^	0.12 ± 0.001 ^g h, A^	0.04 ± 0.001 ^d, A^	7.05 ± 0.001 ^c d e f g, A^	12.46 ± 0.19 ^k, A^
**P-Rice**	2.28 ± 0.10 ^a b c, A^	0.06 ± 0.001 ^a b, A^	0.02 ± 0.001 ^a b, A^	12.43 ± 0.001 ^h, A^	4.57 ± 0.25 ^b c, A^
**P-Commercial rice**	2.50 ± 0.12 ^b c d, A^	0.08 ± 0.001 ^e, A^	0.02 ± 0.001 ^a b, A^	2.78 ± 0.001 ^a b, A^	4.38 ± 0.42 ^a b, A^
**PC-20.0**	2.35 ± 0.20 ^b c, B^	0.09 ± 0.001 ^e f, B^	0.02 ± 0.001 ^a b, B^	1.77 ± 0.001 ^a, B^	5.12 ± 0.54 ^c d, A^
**PC-20.10**	4.47 ± 0.07 ^g, B^	0.13 ± 0.001 ^h i, A^	0.09 ± 0.001 ^f, B^	3.09 ± 0.001 ^a b c, B^	8.20 ± 0.74 ^g h, A^
**PC-40.0**	3.52 ± 0.22 ^f, B^	0.07 ± 0.001 ^c d, B^	0.02 ± 0.001 ^a b, B^	3.89 ± 0.01 ^a b c, B^	5.97 ± 0.36 ^f, A^
**PC-40.10**	4.59 ± 0.25 ^g h, B^	0.13 ± 0.001 ^h i, A^	0.08 ± 0.001 ^f, A^	2.91 ± 0.01 ^a b c, B^	10.01 ± 0.62 ^h i, A^
**PC-60.0**	3.21 ± 0.27 ^e f, B^	0.06 ± 0.001 ^c d, B^	0.02 ± 0.001 ^a b, B^	1.37 ± 0.001 ^a, B^	8.77 ± 0.86 ^g h, A^
**PC-60.10**	5.03 ± 0.26 ^h i j, B^	0.14 ± 0.001 ^j, A^	0.10 ± 0.001 ^g, A^	0.91 ± 0.001 ^a, B^	10.32 ± 1.22 ^k, A^
**PC-80.0**	3.29 ± 0.20 ^e f, B^	0.09 ± 0.001 ^f, B^	0.03 ± 0.001 ^c, B^	5.33 ± 0.001 ^a b c d e, A^	8.09 ± 0.72 ^j, A^
**PC-80.10**	5.83 ± 0.55 ^k l, B^	0.14 ± 0.001 ^i, B^	0.07 ± 0.001 ^e, B^	6.41 ± 0.001 ^b c d e f, A^	9.31 ± 0.60 ^l, A^
**PC-Bean**	2.66 ± 0.18 ^c d, B^	0.06 ± 0.001 ^b c, B^	0.01 ± 0.001 ^a, B^	3.22 ± 0.001 ^a b c, A^	6.99 ± 0.65 ^k, A^
**PC-Rice**	2.11 ± 0.06 ^a b, A^	0.05 ± 0.001 ^a, A^	0.02 ± 0.001 ^a b, A^	13.19 ± 0.001 ^i, B^	4.66 ± 0.74 ^b c, A^
**PC-Commercial rice**	1.80 ± 0.07 ^a, B^	0.07 ± 0.001 ^d, A^	0.01 ± 0.001 ^a, A^	2.63 ± 0.001 ^a b c d, A^	3.08 ± 0.51 ^a b, A^

Values are means ± standard deviation (*n* = 4). Mean values in the same column followed by a different superscript letter are significantly different (*p* < 0.05); small superscript letters mean differences among all of the samples analyzed, whereas capital superscript letters mean differences due to the cooking treatment for the same formulation. C3GE, cyanidin-3-glucoside equivalents; QE, quercetin equivalents; CAE, caffeic acid equivalents; CE, (+) catechin equivalents.

**Table 6:** Effects of the cooking process on the color values in the CIELab space of the uncooked (P-) and cooked (PC-) fettuccine and the commercial rice pasta.

Formulation	Uncooked Pasta (P-)			Cooked Pasta (PC-)		
	L*	a*	b*	L*	a*	b*
**20.0**	84.56 ± 0.21 ^g h, A^	1.42 ± 0.03 ^f, A^	16.62 ± 0.14 ^h, A^	65.03 ± 1.30 ^d, B^	–1.57 ± 0.17 ^a b, B^	12.37 ± 1.26 ^c, B^
**20.10**	69.31 ± 0.15 ^e, A^	4.58 ± 0.07 ^j, A^	14.59 ± 0.17 ^e f, A^	44.44 ± 1.42 ^a, B^	7.37 ± 0.63 ^m, B^	12.60 ± 0.78 ^c d, B^
**40.0**	84.98 ± 0.12 ^h, A^	1.72 ± 0.05 ^f g, A^	18.16 ± 0.23 ^i, A^	65.69 ± 0.61 ^d, B^	–1.27 ± 0.21 ^b c, B^	15.00 ± 0.76 ^f, B^
**40.10**	70.07 ± 0.12 ^e, A^	4.27 ± 0.06 ^j, A^	15.39 ± 0.12 ^f g, A^	45.73 ± 1.17 ^a b, B^	6.42 ± 0.47 ^l, B^	12.56 ± 0.42 ^c d, B^
**60.0**	83.25 ± 0.28 ^f g, A^	2.51 ± 0.06 ^h, A^	20.56 ± 0.44 ^j, A^	65.63 ± 1.08 ^d, B^	–1.04 ± 0.30 ^c, B^	16.23 ± 1.19 ^g h, B^
**60.10**	69.05 ± 0.29 ^e, A^	4.23 ± 0.01 ^j, A^	15.41 ± 0.11 ^f g, A^	47.06 ± 1.48 ^b, B^	5.68 ± 0.50 ^k, B^	11.58 ± 0.63 ^b c, B^
**80.0**	83.66 ± 0.12 ^g h, A^	2.12 ± 0.03 ^g h, A^	19.58 ± 0.08 ^i, A^	65.43 ± 1.19 ^d, B^	–0.50 ± 0.4 ^d, B^	17.77 ± 1.24 ^i, B^
**80.10**	68.44 ± 0.27 ^e, A^	4.69 ± 0.08 ^j, A^	18.29 ± 0.27 ^j, A^	46.26 ± 1.04 ^b, B^	5.84 ± 0.52 ^k, B^	13.63 ± 0.63 ^d e, B^
**Bean 100%**	81.93 ± 0.16 ^f, A^	3.44 ± 0.03 ^i, A^	22.85 ± 0.14 ^k, A^	64.53 ± 1.20 ^d, B^	0.63 ± 0.35 ^e, B^	19.98 ± 0.61 ^j, B^
**Rice 100%**	87.02 ± 0.10 ^i, A^	0.69 ± 0.01 ^e, A^	11.55 ± 0.05 ^b c, A^	62.93 ± 2.09 ^c, B^	–1.86 ± 0.11 ^a, B^	4.42 ± 0.61 ^a, B^
**Commercial rice**	92.01 ± 0.05 ^j, A^	0.30 ± 0.001 ^e, A^	11.07 ± 0.10 ^b, A^	70.08 ± 1.36 ^e, B^	–1.60 ± 0.19 ^a b, B^	12.66 ± 1.54 ^c d, B^

Values are means ± standard deviation (*n* = 12). Mean values in the same column followed by a different superscript letter are significantly different (*p* < 0.05); small superscript letters mean differences among all of the samples analyzed, whereas capital superscript letters mean differences due to the cooking treatment for the same formulation. L*, luminosity; a*, redness/greenness; b*, yellowness/blueness.

**Table 7:** Instrumental measure of the texture of the cooked fettuccine (PC-) and the commercial pasta (control).

	Hardness	Stickiness	Adhesiveness	Cutting	
Sample	(N)	(N)	(N·s)	Firmness (N)	Consistency (N·s)
**PC-20.0**	58.31 ± 0.62 ^d^	9.21 ± 0.19 ^a^	–0.44 ± 0.12 ^a^	3.14 ± 0.04 ^d e^	5.39 ± 0.06 ^b^
**PC-20.10**	44.49 ± 0.44 ^b^	6.66 ± 0.16 ^b c^	–0.41 ± 0.09 ^a b c^	3.14 ± 0.02 ^d e^	5.39 ± 0.04 ^b^
**PC-40.0**	55.56 ± 0.21 ^c d^	6.96 ± 0.20 ^b c^	–0.39 ± 0.11 ^a b c^	3.23 ± 0.02 ^d e^	6.17 ± 0.05 ^c d^
**PC-40.10**	53.51 ± 0.25 ^c d^	5.88 ± 0.18 ^c d^	–0.35 ± 0.07 ^b c d^	4.02 ± 0.08 ^f^	5.49± 0.10 ^b c^
**PC-60.0**	58.21 ± 0.37 ^d^	4.61 ± 0.12 ^d^	–0.30 ± 0.06 ^d^	3.33 ± 0.04 ^e^	6.47 ± 0.08 ^d^
**PC-60.10**	53.21 ± 0.42 ^c^	6.47 ± 0.12 ^c^	–0.40 ± 0.05 ^a b c^	2.74 ± 0.04 ^b c^	5.29 ± 0.09 ^b^
**PC-80.0**	63.50 ± 0.46 ^e^	7.94 ± 0.14 ^a b^	–0.45 ± 0.07 ^a^	4.02 ± 0.03 ^f^	8.13 ± 0.09 ^e^
**PC-80.10**	68.21± 0.70 ^f^	6.17 ± 0.13 ^c^	–0.42 ± 0.06 ^a b^	2.94 ± 0.07 ^c d^	6.66± 0.09 ^d^
**PC-Bean**	70.36 ± 0.65 ^f^	4.51 ± 0.15 ^d^	–0.32 ± 0.09 ^c d^	4.70 ± 0.03 ^g^	10.58 ± 0.15 ^f^
**PC-Rice**	35.28 ± 1.17 ^a^	9.11 ± 0.35 ^a^	–0.39 ± 0.10 ^a b^	1.96 ± 0.04 ^a^	3.53 ± 0.08 ^a^
**PC-Commercial**	31.26 ± 0.32 ^a^	2.74 ± 0.04 ^e^	–0.37 ± 0.09 ^a b c d^	2.45 ± 0.03 ^b^	8.53± 0.11 ^e^

Values are means ± standard deviation (*n* = 12). Mean values in the same column followed by a different superscript letter are significantly different (*p* < 0.05).

## Supplementary Materials

The following are available online at https://www.mdpi.com/2304-8158/9/4/415/s1. Table S1: Correlation coefficients between the phenolic compounds and the antioxidant activity; Table S2: Correlation coefficients between the bioactive compounds and the instrumental texture parameters of cooked fettuccine; Figure S1: The uncooked experimental rice/bean fettuccine without (20.0, 40.0, 60.0, 80.0, bean 100%, rice 100%) and with (20.10, 40.10, 60.10, and 80.10) whole carob fruit and the commercial rice pasta (control); Figure S2: Principal components analysis (PCA) projection of the PC1 and PC2 principal components. P-, uncooked pasta; PC-, cooked pasta. Parameters: Total inositol phosphates (IP Total), total α-galactosides, sucrose, protease inhibitors (TIU and CIU), lectins, total phenolics, tartaric esters, flavonols, anthocyanins, and antioxidant capacity (ORAC); Figure S3: Principal components analysis (PCA) projection of the PC1, PC2, and PC3 principal components. P-, uncooked pasta; PC-, cooked pasta. Parameters: Total inositol phosphates (IP Total), total α-galactosides, sucrose, protease inhibitors (TIU and CIU), lectins, total phenolics, tartaric esters, flavonols, anthocyanins, and antioxidant capacity (ORAC).
