# Supplementary material for: Cooking Effect on the Bioactive Compounds, Texture, and Color Properties of Cold-Extruded Rice/Bean-Based Pasta Supplemented with Whole Carob Fruit
Source: Foods. 2020 Apr 2;9(4):415. doi: 10.3390/foods9040415 (PMC7230172; doi:10.3390/foods9040415)
Supplement: Supplementary file 1 [file foods-09-00415-s001.zip › Suplementary PCA. Figure S2 and S3.pdf]

Principal Component Analysis (PCA)

The principal component analysis (PCA) was carried out on the results obtained of the different bioactive compounds analysed (IP total, Sucrose, Total α-galactosides, Trypsin and Chymotrypsin inhibitors, Lectins, Total phenols, Tartaric esters, Flavonols, Anthocyanins and antioxidant activity (ORAC)) in the ten experimental rice-based fettuccine and in the commercial rice pasta studied as control, uncooked (P-) and cooked (PC-). The data of the all the samples were used to elaborated the principal component analysis.

The first three principal components (PC1, PC2 and PC3) explained the 84.29% of the variance of the original dates. PC1 displayed more than 51% of the systematic variation in the data. The PC2 represent the 20.76% extra variation and the PC3 explain another 11.74% of the variance. The first two principal components were represented in Figure S2. They explained a 72.56% of the variance. PC1 was most correlated with IP total, total galactosides, tartaric esters and antioxidant activity, and PC2 was more correlated with protease inhibitors and flavonols. On other hand, PC3 was correlated with lectins and anthocyanins content.

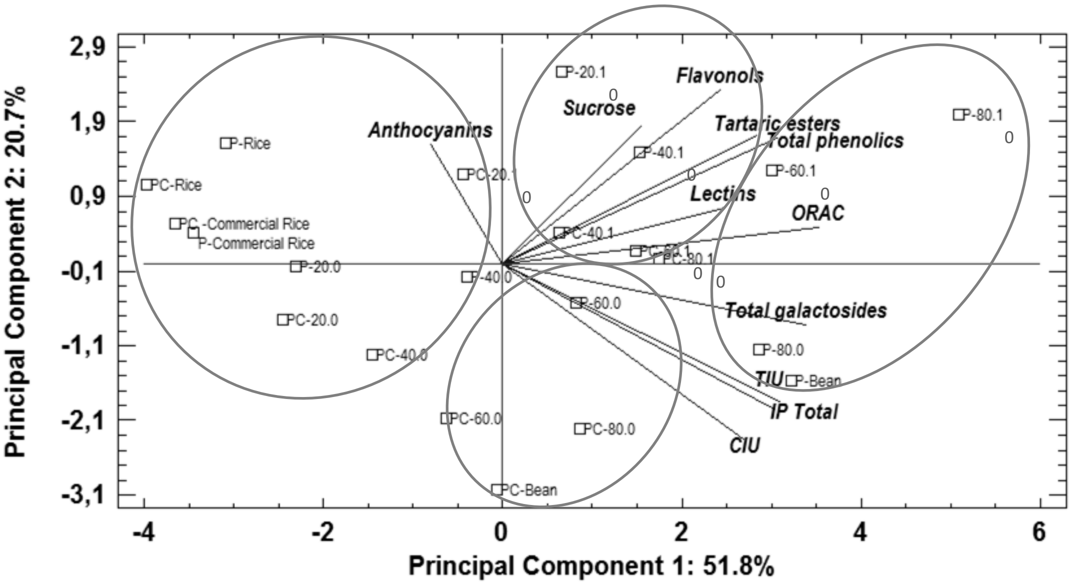

Figure S2. Principal components analysis (PCA) projection of the PC1 and PC2 principal components. P- (uncooked pasta), PC- (cooked pasta). Parameters: Total inositol phosphates (IP Total), Total α-galactosides, Sucrose, Protease inhibitors (TIU and CIU), Lectins, Total Phenolics, Tartaric esters, Flavonols, Anthocyanins and Antioxidant capacity (ORAC).

The differentiation between uncooked (P-) and cooked (PC-) fettuccine was relatively strong between PC1 and PC2. As it can be observed in the Figure S2, the uncooked fettuccine were characterised positively by PC2, and cooked pasta were characterised negatively by the same component (PC2). It is clear from the plot in Figure S1 that the different formulations were not mixed and PC1 separated out well the formulation rice/bean/carob in the samples. The uncooked fettuccine with higher legumes content were characterised positively by PC1 and PC2, while, the group of the uncooked samples with lower legumes content 0% to 50% (P-Rice, P-Commercial, P-20.0, P-20.10, P-40.0 and P-40.10), were represented negatively by the PC1 and positively by

PC2. The uncooked (P-) samples with higher amount of legumes were located mainly in the positive area of both components (PC1 and PC2). The cooked (PC-) samples are located in the negative section of the PC2 and in function of the percentage of legumes in the PC1; the pasta with lower content of legumes (PC-Rice, PC-Commercial Rice, PC-20.0, PC-20.10, PC-40.0 and PC-40.10) were represented negatively by PC1, whereas the PC-60.0, PC-60.10, PC-80.0, PC-80.10, PC-Bean were characterised slightly in the positive area the PC2.

We can have concluded that PC1 characterise the experimental fettuccine with the criterion of the percentage of legumes. The principal component analysis can separate the experimental fettuccine analysed in four broad groups of samples (Figure S2). The first group was formed by the cooked samples with lower legumes content (0-50%); the second group was the cooked samples with 60-100% legumes and characterised by flavonols, tartaric esters, total phenolic, lectins and antioxidant activity; the third group corresponded to the uncooked samples with 0% to 50% of legumes, and finally the last group was integrated by the uncooked samples with high legumes content (60%-100%) and characterised strongly by the parameters: total galactosides, protease inhibitors and total IP.

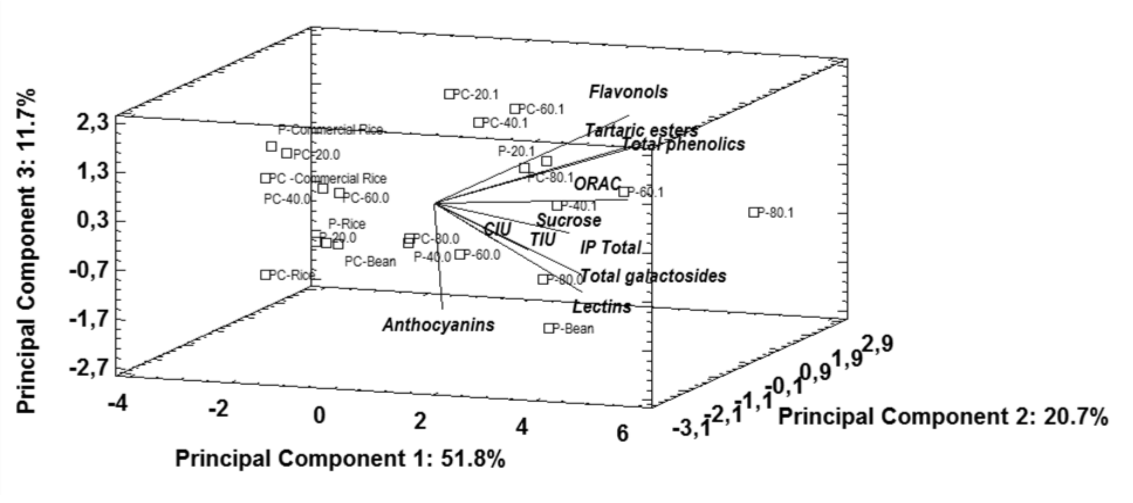

Figure S3. Principal components analysis (PCA) projection of the PC1, PC2 and PC3 principal components. P- (uncooked pasta), PC- (cooked pasta). Parameters: Total inositol phosphates (IP Total), Total α-galactosides, Sucrose, Protease inhibitors (TIU and CIU), Lectins, Total Phenolics, Tartaric esters, Flavonols, Anthocyanins and Antioxidant capacity (ORAC).
