# Supplementary material for: Cooking Effect on the Bioactive Compounds, Texture, and Color Properties of Cold-Extruded Rice/Bean-Based Pasta Supplemented with Whole Carob Fruit
Source: Foods. 2020 Apr 2;9(4):415. doi: 10.3390/foods9040415 (PMC7230172; doi:10.3390/foods9040415)

Figure S1. The uncooked experimental rice/bean fettuccine without (20.0, 40.0, 60.0, 80.0, bean 100%, rice 100%) and with (20.10, 40.10, 60.10 and 80.10) whole carob fruit and the commercial rice pasta (control).

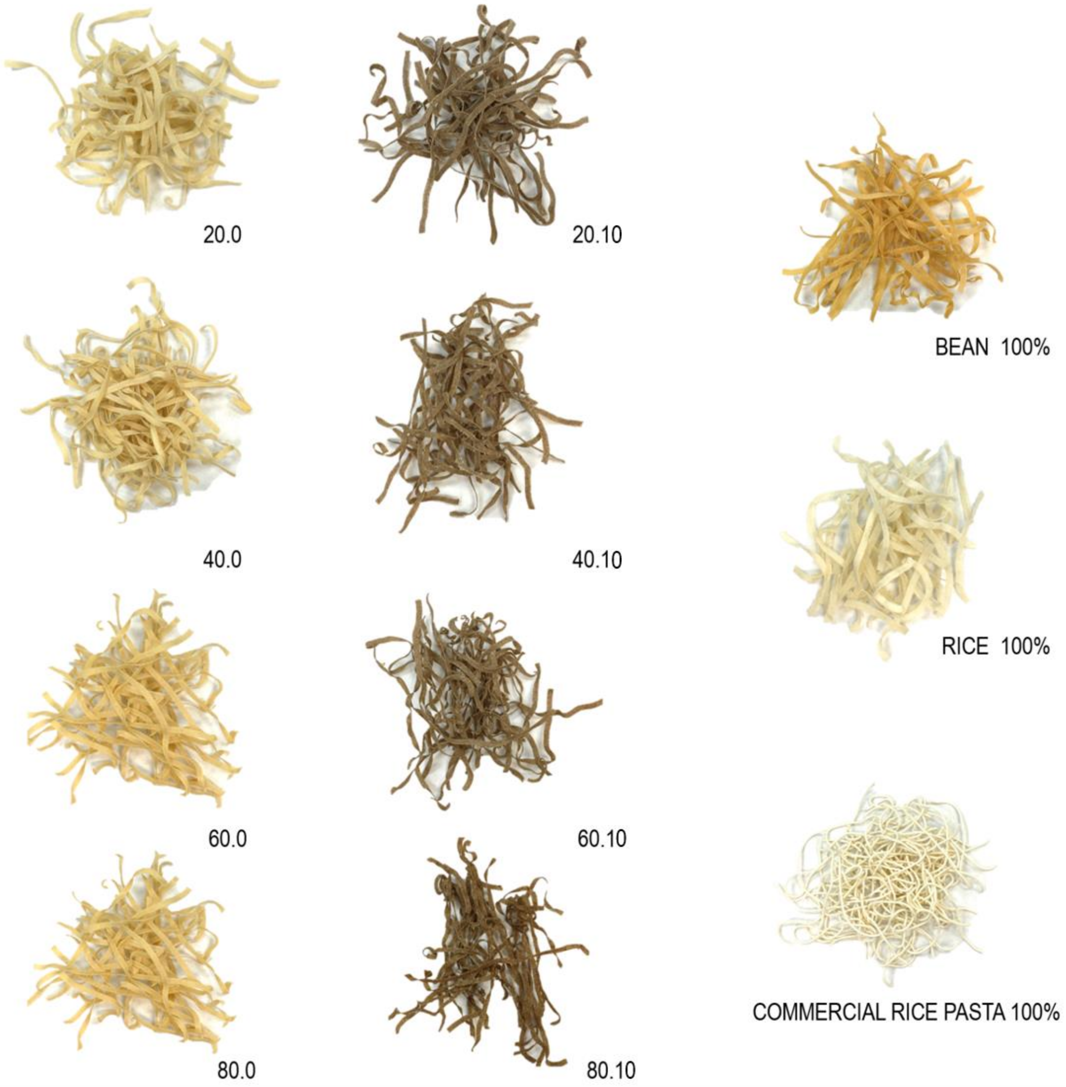

Supplement: Supplementary file 1 [file foods-09-00415-s001.zip › Supplementary Figure S1. Pictures fettuccine and commercial pasta.pdf]
