# Supplementary material for: Cooking Effect on the Bioactive Compounds, Texture, and Color Properties of Cold-Extruded Rice/Bean-Based Pasta Supplemented with Whole Carob Fruit
Source: Foods. 2020 Apr 2;9(4):415. doi: 10.3390/foods9040415 (PMC7230172; doi:10.3390/foods9040415)
Supplement: Supplementary file 1 [file foods-09-00415-s001.zip › Supplementary Table S1. Correlation coeficients Phenols-ORAC.pdf]

Table S1. Correlation coefficients between the phenolic compounds and the antioxidant activity

	Total phenols	Tartaric esters	Flavonols	Anthocyanins
**ORAC**	**0,8852**<br>p = 0,0000	**0,8393**<br>p = 0,0000	**0,7305**<br>p = 0,0000	-0,1255<br>p = 0,1582
