# Supplementary material for: Cooking Effect on the Bioactive Compounds, Texture, and Color Properties of Cold-Extruded Rice/Bean-Based Pasta Supplemented with Whole Carob Fruit
Source: Foods. 2020 Apr 2;9(4):415. doi: 10.3390/foods9040415 (PMC7230172; doi:10.3390/foods9040415)
Supplement: Supplementary file 1 [file foods-09-00415-s001.zip › Supplementary Table S2. Correlation coeficients texture-bioactive compounds.pdf]

Table S2. Correlation coefficients between the bioactive compounds and the instrumental texture parameters of cooked fettuccine.

	Hardness	Stichiness	Adhesiveness	Firmness	Consistency
**Total inositol phosphates**	0,84<br>p = 0,0000	-0,07<br>p = 0,6555	0,05<br>p= 0,7708	0,58<br>p = 0,0000	0,25<br>p = 0,1059
**Total galactosides**	0,73<br>p = 0,0000	-0,10<br>p = 0,502	-0,02<br>p = 0,8736	0,57<br>p = 0,0001	0,29<br>p = 0,0561
**Trypsin inhibitors**	0,81<br>p = 0,0000	0,14<br>p = 0,283	0,15<br>p = 0,2373	0,63<br>p = 0,0000	0,44<br>p = 0,0003
**Chymotrypsin inhibitors**	0,59<br>p = 0,0000	0,10<br>p = 0,4605	0,06<br>p = 0,6547	0,47<br>p = 0,0004	0,31<br>p = 0,0272
**Total phenols**	0,33<br>p = 0,0068	-0,06<br>p = 0,6122	-0,10<br>p = 0,4318	0,02<br>p = 0,8896	-0,24<br>p = 0,0507
